# Functional and Activity Analysis of Cattle *UCP3* Promoter with MRFs-Related Factors

**DOI:** 10.3390/ijms17050682

**Published:** 2016-05-05

**Authors:** Wei Chen, Houqiang Xu, Xiang Chen, Zhongwei Liu, Wen Zhang, Dan Xia

**Affiliations:** 1 Key Laboratory of Animal Genetics, Breeding and Reproduction in the Plateau Mountainous Region, Guizhou University, Guiyang 550025, China; chenweigzu@163.com (W.C.); as.xchen@gzu.edu.cn (X.C.); aszhangwen@sina.com (W.Z.); xiashuang129@163.com (D.X.); 2 College of Animal Science, Guizhou University, Guiyang 550025, China; 3 College of Life Science, Guizhou University, Guiyang 550025, China; ls.zwliu@gzu.edu.cn

**Keywords:** uncoupling protein 3 (*UCP3*), promoter, transcriptional activity, MRFs family, myocyte-specific enhancer factor 2A (*MEF2A*)

## Abstract

Uncoupling protein 3 (*UCP3*) is mainly expressed in muscle. It plays an important role in muscle, but less research on the regulation of cattle *UCP3* has been performed. In order to elucidate whether cattle *UCP3* can be regulated by muscle-related factors, deletion of cattle *UCP3* promoter was amplified and cloned into pGL3-basic, pGL3-promoter and PEGFP-N3 vector, respectively, then transfected into C2C12 myoblasts cells and *UCP3* promoter activity was measured using the dual-Luciferase reporter assay system. The results showed that there is some negative-regulatory element from −620 to −433 bp, and there is some positive-regulatory element between −433 and −385 bp. The fragment (1.08 kb) of *UCP3* promoter was cotransfected with muscle-related transcription factor myogenic regulatory factors (MRFs) and myocyte-specific enhancer factor 2A (*MEF2A*). We found that *UCP3* promoter could be upregulated by *Myf5*, *Myf6* and *MyoD* and downregulated by *MyoG* and *MEF2A*.

## 1. Introduction

Uncoupling protein 3 (*UCP3*) is a member of the family of uncoupling proteins (UCPs), which are members of the mitochondrial anion carrier family. UCPs consist of seven members: *UCP1*, *UCP2*, *UCP3*, *UCP4*, *BMCP1* (brainmitochondrial carrier protein 1), *StUCP* (plant UCP homologs have been identified in *Solanum tuberosum*) and *AtUCP* (*Arabidopsis thaliana*) [1,2]. *UCP3* was first discovered and described in 1997 [3,4]. Because of its close homology with *UCP1, UCP3* was initially implicated in thermoregulation [3,4], as it has been demonstrated to uncouple in a number of experimental models. Uncoupling protein-3 (*UCP3*) gene is primarily expressed in skeletal muscle and up-regulated by fatty acids [5]. *UCP3* is primarily expressed in skeletal muscle, but is also found in brown fat and heart tissue [6]. *UCP3* protein is one of the most important target proteins involved in skeletal muscle energy metabolism substance, and plays key regulatory roles in mitochondrial fatty acid oxidation [7,8,9,10,11]. Until recently, the eukaryotic core promoter recognition complex played a critical regulatory role in driving cell specific programs of transcription during development [12]. Several studies have shown that a transcription factor not only plays a control function in gene expression, but also inhibits the action of the complex combination [13]. *MyoD* controls *UCP3* promoter activity through a noncanonical E box site located close to the transcription initiation site [5]; in this context, it is important to note that the *MyoD* response element is located at different positions in rats compared to mice/humans, which may cause species-specific differences in *UCP3* expression [14]. The study of the molecular mechanisms of this transcription element have shown that it can synthesize muscle specific activity of the promoter, some promoters have been synthesized that have high expressing activity specificity in the muscle. With continuous development and improvement of the research, the regulation of muscle-specific promoter element values to control muscle-specific promoter elements will become a powerful tool for animal muscle development research [15].

Guanling cattle are a yellow-coated breed that was developed in China [16]. In this study, 5′ upstream regions of *UCP3* of Guanling cattle were analyzed, and PCR amplified segments of different length *UCP3* gene promoter sequences, different length fragments of *UCP3* promoter activity and the core of *UCP3* promoter were tested. Transcription factor binding sites of cattle *UCP3* promoter were screened using Promoter-Binding transcription factors (TF) Profiling assay combined with bioinformatics analysis, and we identified several transcription factor binding sites for *UCP3*. We investigated whether the *UCP3* promoter activity could be increased by myogenic regulatory factors (MRFs) family and myocyte-specific enhancer factor 2A (*MEF2A*) transcriptional factors. These results of cattle *UCP3* promoter provide a platform for further researche on promoter activity and expression regulation. The results are meaningful in the research of muscle-specific promoters and the mechanism of cattle’s muscle development and growth.

## 2. Results

### 2.1. Analysis of Uncoupling Protein 3 (UCP3) Gene Using qRT-PCR

**Figure 1:**
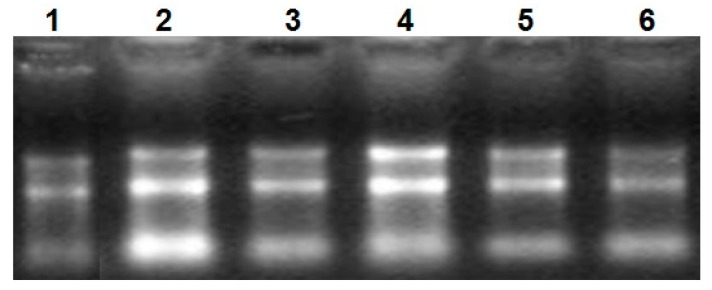
The electrophoretogram of RNA. From1 to 6, the results represent heart tissue, liver tissue, small intestine tissue, longissimus dorsi tissue, hind shin tissue, adipose tissue, respectively.

**Figure 2:**
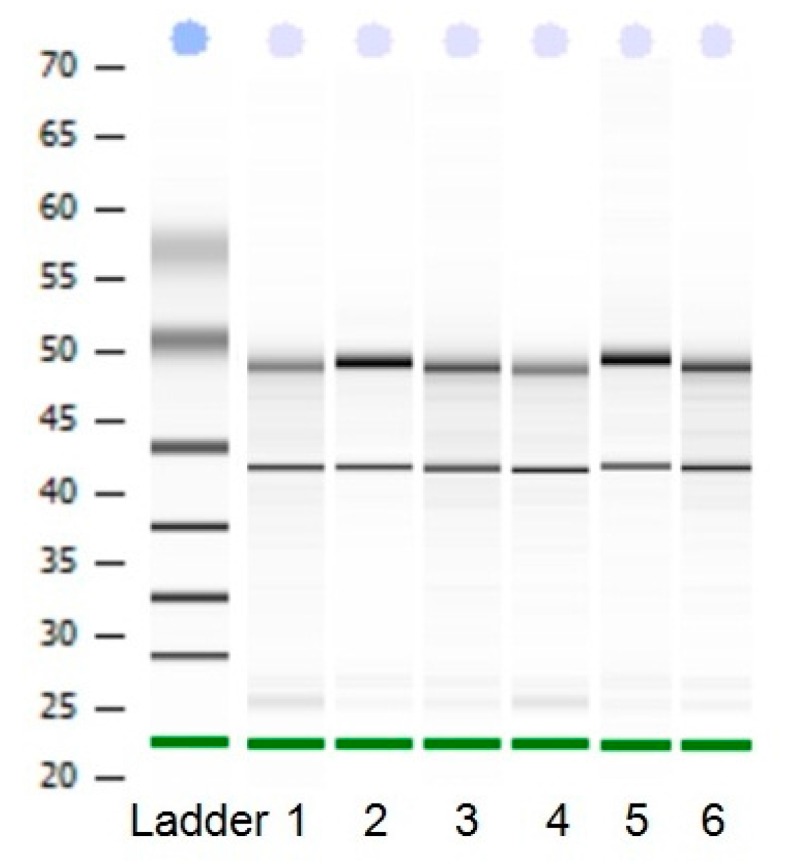
The 2100 biological analyzer results of RNA. From1 to 6, the results represent heart tissue, liver tissue, small intestine, longissimus dorsi, hind shin, adipose tissue, respectively.

**Figure 3:**
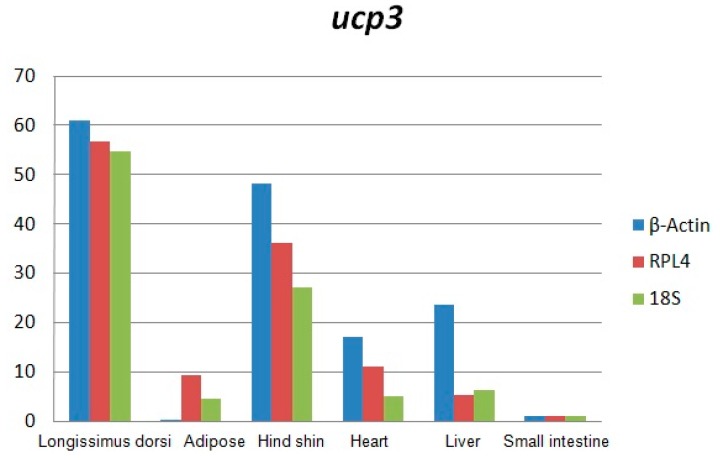
The expressing level of uncoupling protein 3 (*ucp3*) gene in Guanling cattle different tissues.

Total RNA was analyzed by 2100 expert_Eukaryote Total RNA Nano and Agarose gel electrophoresis (Figure 1 and Figure 2). The results showed that purity, number and integrity of RNA samples conformed to the requirements of the experiments. The levels of *UCP3* gene expression in the longissimus dorsi, adipose tissue, hind shin, heart, liver, and small intestine tissues were recorded using qRT-PCR. The result showed that expression of the *UCP3* was at a high level in longissimus dorsi and hind shin tissues. The lowest level was in small intestine (Figure 3), and the results showed consistent expression of β-actin, RPL4 and 18S RNA in six tissues. The qRT-PCR data confirmed that the ucp3 was tissue-specifically expressed in the longissimus dorsi and hind shin.

### 2.2. Cloning and Activity Analysis of the Cattle ucp3 Promoter

**Figure 4:**
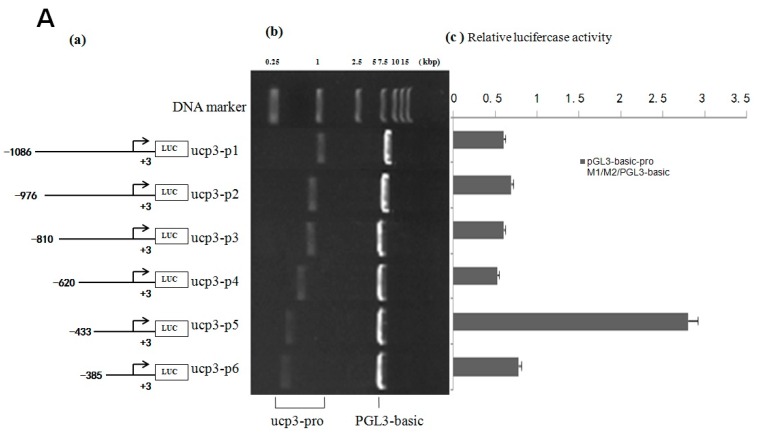

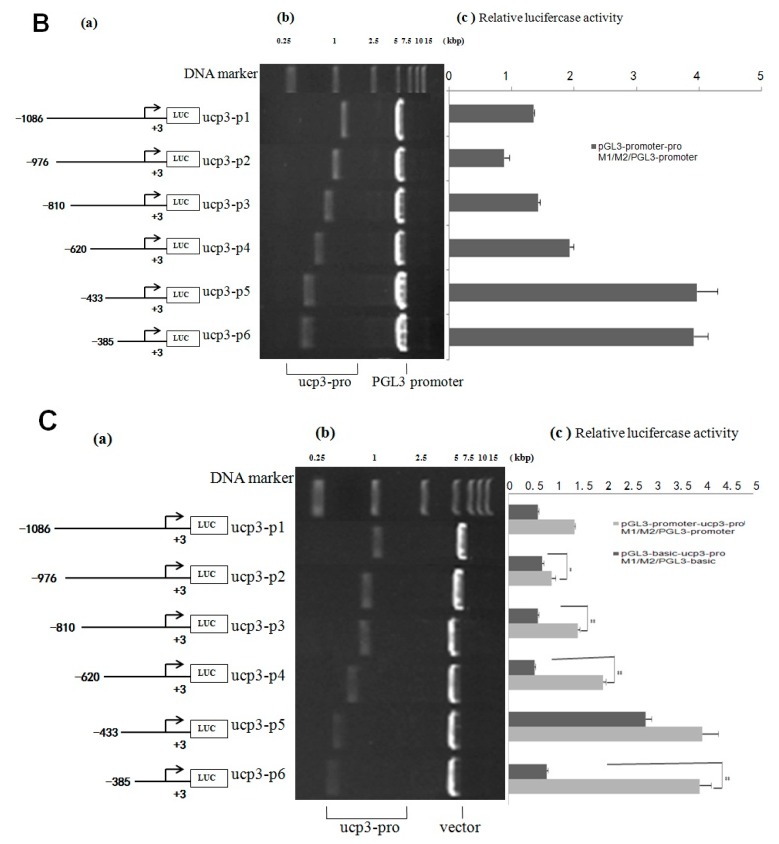
Deletion analysis of the cattle *UCP3* promoter. (**A**) Promoter activity of the pGL3-basic-ucp3-Pro in C2C12 Cells; (**B**) Promoter activity of the pGL3-promoter-ucp3-Pro in C2C12 Cells; (**C**) Activity of the ucp3 promoter in C2C12 Cells. (**a**) Schematic diagram of the *UCP3* promoter constructs consisting of the 5′-flanking region with serial deletions cloned into the pGL3-basic and pGL3-promoter vector. The arrows show the direction of transcription. The numbers represent the end points of each construct; (**b**) The deletion plasmids were digested by restriction enzyme and run on a 1.5% agarose gel. The size of the vector is 4.8 kb. The inserted fragments of the *UCP3* promoter range from 389 to 1086 bp which are confirmed by sequencing; (**c**) The deletion plasmids were cotransfected with pGL3-basic and pGL3-promoter into C2C12 cells; the cells were harvested 24 h later after transfection and luciferase activity was measured and expressed in relative luciferase units (RLU). The values represent means ± SE. vector represents pGL3-basic, *n* = 3, ** *p* < 0.01, by analysis of variance with one-way ANOVA.

**Figure 5:**
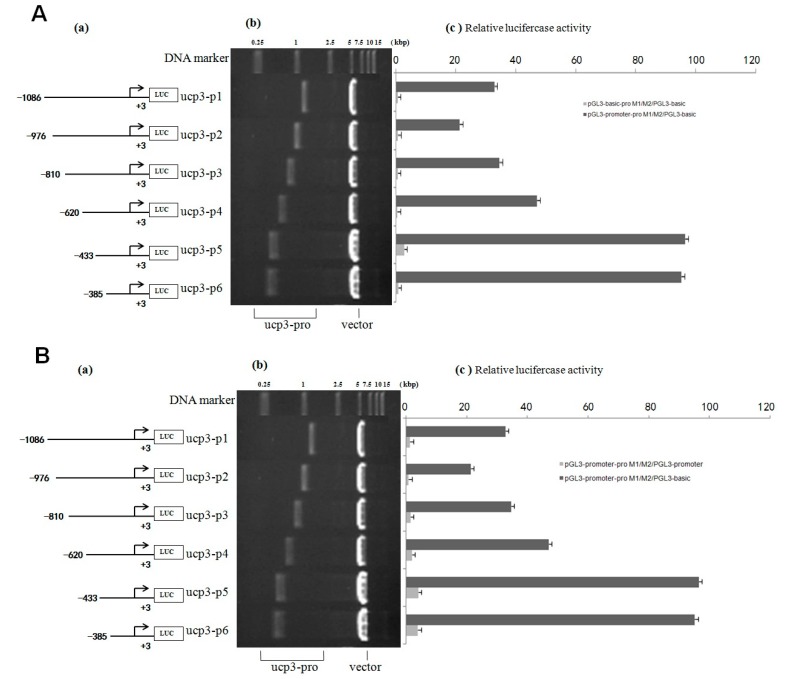
Analysis of the cattle *UCP3* promoter. (**A**) Analysis of activity of pGL3-basic-ucp3-pro/pGL3-basic and pGL3-promoter-ucp3-pro/pGL3-basic; (**B**) Analysis of activity of of pGL3-promoter-ucp3-pro/pGL3-basic and pGL3-promoter-ucp3-pro/pGL3-promoter. (**a**) Schematic diagram of the ucp3 promoter constructs consisting of the 5′-flanking region with serial deletions cloned into the pGL3-basic and pGL3-promoter vector. The arrows show the direction of transcription. The numbers represent the end points of each construct; (**b**) The deletion plasmids were digested by the restriction enzyme and run on a 1.5% agarose gel. The size of vector is 4.8 kb. The inserted fragments of the ucp3 promoter range from 389 to 1086 bp which are confirmed by sequencing; (**c**) The deletion plasmids were cotransfected with pGL3-basic and pGL3-promoter into C2C12 cells; the cells were harvested 24 h later after transfection and luciferase activity was measured and expressed in relative luciferase units (RLU).

The result shows that the eukaryotic expression vectors pGL3-basic-ucp3-pro and pGL3-promoter-ucp3-pro were constructed successfully (Figure 4 and Figure 5). The promoter activities of the different deleted regions were measured in C2C12 myoblasts cells. A series of pGL3-basic-ucp3-pro and pGL3-promoter-ucp3-pro recombinant vectors with different fragmens of *UCP3* promoter were constructed (Figure 4 and Figure 5) to determine the minimal promoter region of *UCP3* promoter. The results indicate that the deletion plasmid pGL3-basic-ucp3-P5 containing the promoter region from −433 to +3 bp and the plasmid pGL3-ucp3-P6 containing the promoter region from −385 to +3 bp had significant activity compared to pGL3-basic. These results suggest that the sequence from −385 to +3 bp has the minimal regulatory region that contains the transcription start site necessary for ucp3 promoter activity (Figure 4A,B). The 5′-upstream region from −433 to +3 bp of the *UCP3* promoter only induced 4-fold increases, which is lower than the increases associated with the pGL3-basic in the luciferase activities. Our data show that the 5′-flanking region of *UCP3* from −385 to +3 bp contains the basal promoter element of the cattle *UCP3*.

pGL3-basic-ucp3-P5 had low promoter activity, but pGL3-promoter-ucp3-p5 and pGL3-promoter-ucp3-p6 exhibited significantly higher promoter activity (96.54 ± 3.96 and 95.25 ± 3.91 relative luciferase units (RLU), relative to pGL3-basic) (Figure 5A,B). This difference in the promoter activities between different segments of *UCP3* promoter suggests that additional factors can increase the promoter activity of *UCP3*. The result showed that the trend of relative luciferase activity units of different pGL3-basic-ucp3-pro/pGL3-basic were similar to those of pGL3-basic/pGL3-basic (Figure 5). In contrast, the relative luciferase activity units of pGL3-promter-ucp3-pro/pGL3-basic increased, with a 20-fold to 100-fold increase observed compared to that for pGL3-promoter-ucp3-pro/pGL3-basic (Figure 5). These results indicate that the additional promoter had a positive affect on the expression of the target gene before *UCP3* promoter.

### 2.3. Expression of PEGFP-N3-ucp3-pro in C2C12 Cell Line

**Figure 6:**
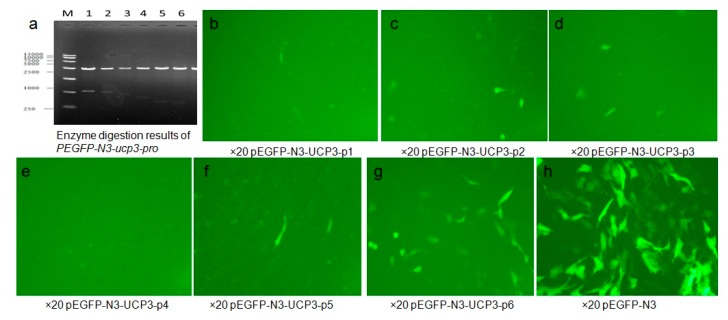
Identification and analysis of PEGFP-N3-ucp3-pro. (**a**) Enzyme digestion results of PEGFP-N3-ucp3-pro recombinant plasmid. M represent 15,000 bp marker, from1 to 6, the results represent PEGFP-N3-ucp3-p1–PEGFP-N3-ucp3-p6, respectively; (**b**–**h**) The green fluorescence of recombinant expression vector PEGFP-N3-ucp3-pro transitional C2C12 cell (Fluorescent inverted microscope ×20).

In this study, plasmid PEGFP-N3-ucp3-pro and PEGFP-N3 transfection C2C12 cells after 48 h were observed with an inverted fluorescence microscope to determine the fluorescence peak of the recombinant plasmid expression in C2C12 cells. The results show that Eukaryotic expression vector PEGFP-N3-ucp3-pro was successfully constructed (Figure 6). Green fluorescent protein were partly expressed in the cytoplasm and nuclei of C2C12 cells, but non-existent in PEGFP-N3-ucp3-P4. Green fluorescence was expressed in various strengths in different segments of the promoter. The result showed that PEGFP-N3 > PEGFP-N3-ucp3-P6 > PEGFP-N3-ucp3-P5 > PEGFP-N3-ucp3-P2 > PEGFP-N3-ucp3-P3 > PEGFP-N3-ucp3-P1 > PEGFP-N3-ucp3-P4. The six promoters that were constructed have differential promoter activity: the promoter activity of P6 and P5 promoter are the highest. The region from −385 to +3 bp contains the functional promoter of the cattle *UCP3* promoter, it is regarded as the core promoter of *UCP3* and is consistent with the result of pGL3-basic-ucp3-pro.

### 2.4. The Screening of Transcription Factor by Promoter-Binding Transcription Factors (TF) Profiling Assay II

**Figure 7:**
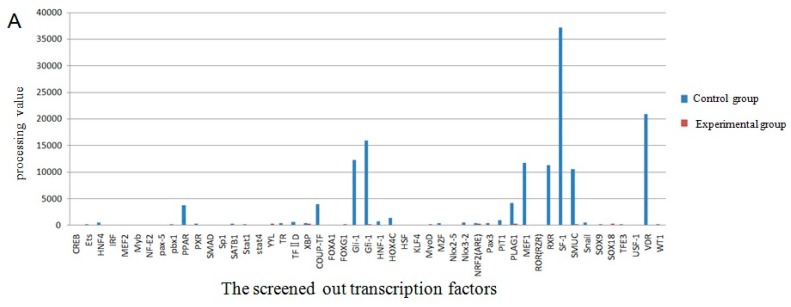

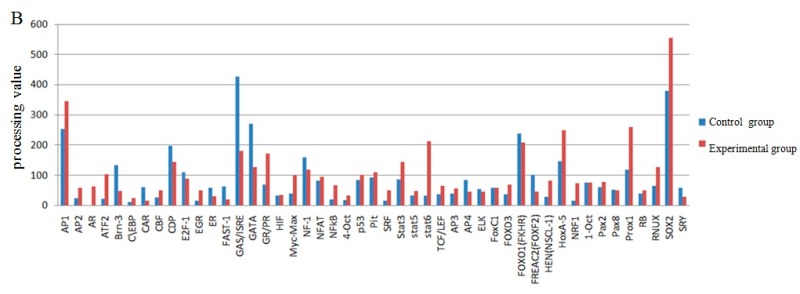
Transcription factor screening results. (**A**) The comparison chart of the control group and experimental group processing value of the screened out transcription factors; (**B**) The comparison chart of the control group and experimental group processing value of some transcription factors.

The screening of transcription factor of *UCP3* promoter using promoter-binding TF profiling Assay II detects the experimental results by multifunctional microplate chemiluminescence detection function. The data obtained by the experimental methods deals with the data analysis processing value. The processing value represents the intensity of luminosity. In essence, it represents the probe number after the nuclear extracts corresponding transcription factor binding probe has been eluted. Indirectly, it indicates whether the promoter region contains the transcription factor binding sites. The results from the data processing point of view could determine that the *UCP3* promoter region had *MEF2*, *MyoD*, *MZF*, *pax-5*, *PPAR*, *SATB1*, *TFIID*, *HOX4C*, *Nkx2-5*, *Nkx3-2*, *Pax3*, *MEF1*, *Snail*, *NRF2* (*ARE*) transcription factor binding sites (Figure 7). The predictions of transcription factor binding sites of Guanling cattle ucp3 promoter region by online software show that it contains *MyoD, MZF1* and *Nkx-2* transcription factor binding sites (Table 1 and Table 2). In summary, Guanling cattle *UCP3* promoter regions had *MyoD*, *TFIID*, *Pax3*, *MEF1*, *MEF2* transcription factor binding sites.

### 2.5. Effect of Transcription Factors on UCP3 Promoter Activity

**Figure 8:**
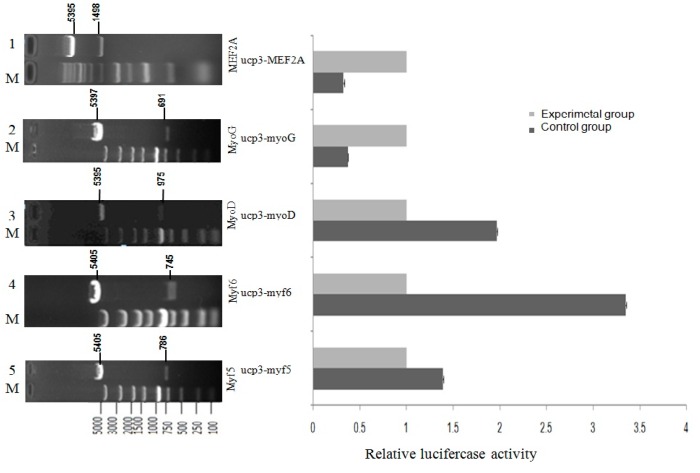
The effect of transcriptional activity of Guangling Cattle *UCP3* promoter by MRFs family and myocyte-specific enhancer factor 2A (*MEF2A*) in C2C12 myoblasts.C2C12 myoblasts were transfected with the reporter gene constructs, pGL3-basic-ucp3-p1, pGL3-Basic, and the internal control, pcDNA3.1(+)-MRFs family and pcDNA3.1(+)-MEF2A expression vector. After 48 h, the cells were harvested for reporter gene assays. The normalized firefly luciferase activity of the experimental group was compared to that of the control group, which was transfected with an empty expression vector containing no cDNA. The control group (open bar) value was set at 1.0, with the fold induction of each group being determined. The bars represent the means ± SE of triplicate determinations.

By design, restriction enzyme sites contain primers and high-fidelity enzymes to enable the cloning of coding area sequence of MRFs family and *MEF2A*, and insert the fragment orientation to the eukaryotic expression vector pcDNA3.1(+) polyclonal loci of the plasmid. Coding area sequence of the MRFs family and *MEF2A* were cloned through double enzyme digestion and sequenced. The result shows that the eukaryotic expression vector pcDNA3.1(+)-MRFs family and pcDNA3.1(+)-MEF2A were constructed successfully (Figure 8), as a base for expression of MRFs family and *MEF2A* transcription factors in the eukaryocyte.

MRFs family and *MEF2A* potential activate an *UCP3* promoter fragment of 1080 bp were detected through dual luciferase reporter gene assay after cotransfection. To evaluate the influence of the MRFs family and *MEF2A* we compared cotransfections with or without expression vectors for *MyoD*, *Myf5*, *Myf6*, *MyoG* and *MEF2A* factors known to regulate *UCP3* promoter expression (Figure 8). In the pGL3-basic-ucp3-p1 transfected group, the pGL3-basic vector showed responsiveness to the co-expression of *Myf5*, *Myf6* or *MyoD* alone respectively. For pGL3-basic-ucp3-p1, the activity increased 1.4-fold more by *Myf5*, 3.3-fold more by *Myf6*, and 2-fold more by *MyoD*, compared to the activities in C2C12 cells transfected with pGL3-Basic.This indicates that the increases in the activity of the *UCP3* promoter are brought about by the transcription factors *Myf5*, *Myf6* and *MyoD*. In contrast, the co-expression of *MyoG* and *MEF2A* resulted in only 0.37- and 0.32-fold changes of luciferase activity, respectively.These results indicate that *MyoG* and *MEF2A* have a negative regulation role of UCP3 promoter.

## 3. Discussion

The results of this study showed that the expression of the *UCP3* was almost undetectable in small intestine and adipose. However, the expression of the *UCP3* in longissimus dorsi and hind shin tissue were about 60-fold and 40-fold greater than the expression of *UCP3* in adipose and the small intestine, and about 6-fold and 4-fold greater than the in liver and heart, suggesting it has characteristics of high efficiency, and specificity expression in skeletal muscle, which was consistent with the observation that the preferential expression of *UCP3* in skeletal muscle and brown fat [3,17,18,19,20] and the *UCP3* promoter being activated in the muscle cell differentiation process [21].

The cloned 5′-upstream regions of the Guanling cattle *UCP3* genes showed varying degrees of promoter activity transfection into C2C12 cells. The result showed that the sequence of *UCP3* promoter from −385 to +3 bp has the minimal promoter region containing the transcription start site necessary for *UCP3* promoter activity, and several studies have reported activity of the *UCP3* promoter in C2C12 [22,23]. Deletion of *UCP3* promoter expression showed that it has some negative-regulatory elements from −620 to −433 bp, and there exist some positive-regulatory elements between −433 and −385 bp. Therefore, this difference in the promoter activities between different segments of *UCP3* promoter suggests that additional factors were needed to activate the *UCP3* promoter. Note that pGL3-basic-ucp3-P5 had much lower promoter activity, but pGL3-promoter-ucp3-p5 and pGL3-promoter-ucp3-p6 exhibited significantly higher promoter activity (96.54 ± 3.96 and 95.25 ± 3.91 RLU, relative to pGL3-basic). In a word, the results showed that additional SV40 promoter can strengthen the activity of *UCP3* promoter, thereby optimizing the question about low activity of *UCP3* promoter in skeletal muscles. The result of PEGFP-N3-ucp3 recombinant vector transfection into C2C12 cells was in agreement with the result of pGL3-promoter-ucp3 transfection into C2C12 cells.

Previous studies had shown that transcription factors combined with these special sequences can open or close a set of specific gene expressions. The interaction of regulatory sequence and transcription factors plays a key role in gene expression [13]. Bioinformatic analysis revealed the presence of some potential transcription factor binding sites of *UCP3* promoters such as *MyoD*, *MZF*, *sox-5*, *TATA*, *Nkx2.* The result of the software also includes *MyoD*, *MZF1* and *Nkx-2.* We have shown that the *UCP3* promoter of Guanling cattle have *MyoD*, *TFIID*, *Pax3*, *MEF1* and *MEF2* transcription factor binding sites by promoter-binding TF brofiling Assay II study. Our experimental and bioinformatics analysis revealed that the Guanling cattle *UCP3* promoter region had *MyoD*, *TFIID*, *Pax3*, *MEF1*, *MEF2* transcription factor binding sites. Several studies have reported that the muscle-specific promoter contained Trex/MEF-3 components, *MEF-1*, *Pax3/7*, *SRE*, *MEF-2*, *CACCC* boxes and other transcriptional regulatory components [14]. Multiple lines of evidence have shown that PPARα and PPARδ are mediators of the fatty acid-dependent control of *UCP3* transcription in skeletal muscle [24]. *Pax3* is a key regulator of *MyoG* during development [25,26]. The embryonic progenitors that express *Pax3*, and its close homolog *Pax7*, give rise to a population of adult muscle stem cells [27,28]. *MEF2* was considered to be a conservative member of the vast majority of muscle-specific genes [29,30,31,32,33]. *MEF2* and the transcription factor containing the basic helix-loop-helix (bHLH) could coordinate muscle genes expression and regulation of the initial myogenic differentiation [34]. In addition, promoter activity was induced by overexpression of *MyoD1*, which bound to this canonical E-box during C2C12 differentiation [35].

To explore the role of *UCP3* promoter for a possible regulation of MRFs family and *MEF2A* transcription factor, *Myf5*, *Myf6*, *MyoD*, *MyoG* and *MEF2A* were selected as the most likely transcription factors to be involved in this study. C2C12 myoblasts were co-transfected with the MRFs family transcription factor or *MEF2A* transcription factors expression vectors and pGL3-basic-ucp3-pro reporter vectors. The results showed that *Myf5*, *Myf6* and *MyoD* contributed to the up-regulation of the *UCP3* expression in C2C12 myoblasts, which was consistent with the present results that *MyoD* was required to activate the promoter of human *UCP3* [5]. Several transcription factor binding sites in the *UCP3* promoter from cattle have been identified including *MyoD*, which is also present in mouse [5] and rat [14]. Conversely, the *MyoG* and *MEF2A* had down-regulation effects on the *UCP3* promoter region. Interestingly, the effects of MRFs family and *MEF2A* were different in C2C12 cell line, *Myf5*, *My6* and *MyoD* increased the dual luciferase activity, but myogenin and *MEF2A* had no effect. These results indicated that both the MRFs family and *MEF2A* are necessary, but, together, they were not sufficient enough transcription factors to induce *UCP3* promoter activity. The result is consistent with previous findings [21].

In summary, the results indicated that this difference was most likely conferred by different ways in which *UCP3* promoter regions respond to the *Myf5*, *Myf6* and *MyoD* transcription factors, and the promoter-binding TF profiling assay shows that these transcription factors bind directly to the *UCP3* promoter. We only focus the *UCP3* promoter in this study, so we can not exclude the possibility that the intoronic region (e.g., intron 1) is associated with the regulation of *UCP3* gene [36,37].

## 4. Experimental Section

### 4.1. Experimental Animals and Tissue Sampling

Guanling cattle (castrated steers, *n* = 3) with similar genetic backgrounds in Guanling county of Guizhou province were reared under the same experimental conditions. At a mean age of 18 months, the animals were slaughtered using standard commercial procedures. We collected longissimus dorsi, adipose tissue, hind shin, heart, liver, and small intestine tissue samples. The tissue samples were placed in the RNAlater RNA stabilization solution (Qiagen, Hilden, Germany) immediately after collection. The samples were frozen in liquid nitrogen, and stored at −80 °C.

### 4.2. RNA Isolation and Synthesis of cDNA

Total RNA was isolated from each tissue sample using the TRIzol reagent (Invitrogen, Waltham, MA, USA), according to the manufacturer’s instructions. An aliquot containing 1 μg of total RNA was used for the synthesis of complementary DNA (cDNA) by reverse transcription using TransStart^®^ One-Step gDNA Removal and cDNA Removal and cDNA Synthesis Super Mix (TransGen Biotech, Beijing, China).

### 4.3. Confirmation of Gene Expression Using qRT-PCR

The primer pairs of *UCP3* were designed to have matching melting temperatures. The primer sequences are listed in Table 3. The same RNA used for *UCP3* analysis of the longissimus dorsi, adipose, hind shin, heart, liver, and small intestine tissue samples were used in the quantitative reverse transcription and polymerase chain reaction (qRT-PCR) experiments. The first strand of the cDNA was synthesized from 1 µg of total RNA using the TransScript II One-Step gDNA Removal and cDNA Synthesis SuperMixkits (TransGen Biotech, Beijing, China) with the oligo (dT) 20 primer (TransGen Biotech). The real-time PCR reactions were performed in a final volume of 20 µL using the Trans Start Green qPCR SuperMix UDG kit (TransGen Biotech), according to the manufacturer’s instructions. The mRNA expression were normalized to that of the β-actin, RPL4 and 18SRNA gene. The real-time PCR assays were performed using a CFX96 real-time PCR system (Bio-Rad, Hercules, CA, USA). Thermal cycling was performed using an initial denaturation step of 95 °C for 1 min, followed by 40 cycles of 95 °C for 30 s, 58 °C for 30 s, and 72 °C for 1 min. A melting curve was constructed using annealing temperatures from 58 to 95 °C to verify the specificity of the amplified product. The data were analyzed using the 2^−ΔΔ*C*t^ method.

### 4.4. Amplify UCP3 Promoter of Guanling Cattle

**Figure 9:**
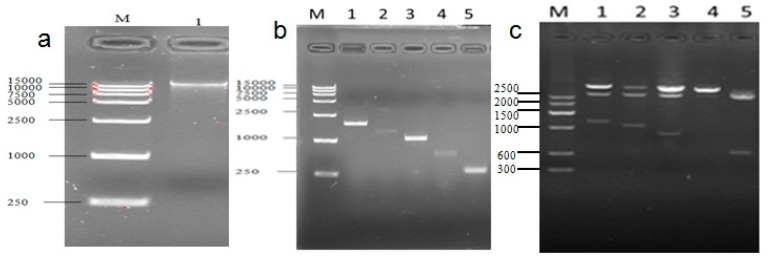
Cloned and identified sequence of the cattle *UCP3* promoter (**a**) The electrophoretogram of DNA, the product was run on a 1.5% agarose gel; (**b**) Different PCR products of *UCP3* promoters; (**c**) Restriction analysis Identification of pUCm-T-ucp3-pro.

Genomic DNA of Guanling cattle was extracted from blood samples using DNA extraction kit (TransGen Biotech, Beijing, China) according to the manufacturer’s instructions. Based on the sequence of UCP3 gene of cattle (UCSC NM_174210), a pair of PCR primers were designed using Primer Premier 5.0 (version 5.00, PREMIER Biosoft international, Palo Alto, CA, USA, 2000) to amplify 1080, 980, 814, 624, 437 and 389 bp sequence of *UCP3* promoter (Table 4). Six fragments of *UCP3* promoter were amplified by PCR form DNA genome of Guanling cattle (Figure 9). The PCR system consisted of 1 µL templates, 1 µL of each antiprimer, 1 µL primer, 10 µL 2× PCR Master Mix (Takara, Dalian, China) and 7 µL ddH2O. Thermal cycling was performed using an initial denaturation step of 94 °C for 3 min, followed by 30 cycles of 94 °C for 30 s, 56–60 °C for 30 s, and 72 °C for 1 min. After the cycles, a final extension was performed at 72 °C to 7 min. The amplified fragments were digested with Nhe I and xho I, and ligated together into pGL3-basic, pGL3-promoter and PEGFP-N3 at NheI and xhoI site. Double enzyme fragment lengths were 1080, 980, 814, 624, 437 and 389 bp, respectively (Figure 5, Figure 6 and Figure 7). The PCR product was ligated into pUCm-T easy vector, pGL3-basic, PGL3-promoter, PEGFP-N3 and sequenced.

### 4.5. Transcription Factor Profiling of UCP3 Promoter by Filter Assay

For monitoring the activation of multiple TFs of the *UCP3* promoter simultaneously, Promoter-Binding TF Profiling Assay II was used according to the protocol provided by Signosis Inc. (Signosis Inc., Santa Clara, CA, USA). Nuclear proteins were isolated from longissimus dorsi using the reagents and protocol provided by a Nuclear Extraction Kit (Signosis Inc., Santa Clara, CA, USA). Androgen receptor (AR) was used to normalize the readings for as a blank control (Table 5). Luminescence was reported as relative light units (RLUs) using a multidetection microplate reader (Bio Tek, Vermont, VT, USA). TFSEARCH (http://diyhpl.us/~bryan/irc/protocol-online/protocol-cache/TFSEARCH.html) and ALGGEN ROMO (http://alggen.lsi.upc.es/) were used to predict the transcription factor binding sites of *UCP3* promoter region.

### 4.6. Construction of Myogenic Regulatory Factors (MRFs) Family and MEF2A Transcription Factors Expression Vectors

RNA was isolated from longissimus muscle tissue of Guanling cattle using Trizol (Invitrogen). First strand cDNA was synthesized by the Reverse transcription kit (TransGen Biotech, Beijing, China). The primers of *MyoD*, *Myf5*, *Myf6*, *MyoG* and *MEF2A* were designed according to the sequence of NCBI were amplified from the cDNA of longissimus dorsi (Table 6). The MRFs family and *MEF2A* fragments were excised with restriction enzyme, which were ligated into the pcDNA3.1 (Promega, Madison, WI, USA). Then they were confirmed by dual-enzyme digestion and sequencing. Recombinant expression vectors were pcDNA3.1(+)-MyoD, pcDNA3.1(+)-Myf5, pcDNA3.1(+)-Myf6, pcDNA3.1(+)-Myogenin and pcDNA3.1(+)-MEF2A respectively.

### 4.7. Analyse Activity of UCP3 Promoter

Purification of *UCP3* promoter fragments was excised with NheI and XhoI restriction enzyme, and ligated into the pGL3-Basic, pGL3-promoter and PEGFP-N3 (Promega, Madison, WI, USA), respectively. Then they were confirmed by dual-enzyme digestion and sequencing.

The C2C12 myoblasts were obtained from the Cell Bank of the Chinese Academy of Sciences (Shanghai, China). C2C12 myoblasts cells were cultured in DMEM (Hyclone, logan, UT, USA) medium with 10% fetal bovine serum (FBS) (Gibco, Aucland, New Zealand) under normal culture conditions (5% CO_2_ at 37 °C). C2C12 myoblasts were seeded into 24-well plates at a density of 1 × 10^5^/well. C2C12 myoblasts reached 80% confluence after 18–24 h. Subsequently, C2C12 myoblasts were cultured in OPTI-MEM (500 µL/well) containing of 2 µL of lipofectamine 2000, 0.8 µg of the pGL3-Basic-ucp3-pro and pGL3-promoter-ucp3-pro recombinant vcectors and 0.06 µg of the internal control vector pRL-TK/luciferase reporter plasmid, C2C12 myoblasts was cultured in OPTI-MEM (500 µL/well), after 5 h, the OPTI-MEM medium was removed and replaced by DMEM medium containing 10% FBS. After 24 h transfection, cells were harvested, luciferase activity was measured using the Dual-Luciferase^®^ Reporter Assay System (Promega, Madison, WI, USA), and then the activities of firefly luciferase in pGL3 and Renilla luciferase in pRL-TK were analyzed.

Cotransfection of eukaryotic expression vectors of *MyoD*, *Myf5*, *Myf6*, *MyoG* and *MEF2A* with *UCP3* promoter, C2C12 cells was seeded at the density of 1 × 10^5^/well into 24-well plates using DMEM containing 10% FBS medium. After 18–24 h, the plated cells were transfected with 0.6 µg of pGL3-basic-ucp3-P1 vector, 0.06 µg of the internal control vector pRL-TK, 0.2 µg of the expression vectors of pcDNA3.1(+)-MyoD, pcDNA3.1(+)-Myf5, pcDNA3.1(+)-Myf6, pcDNA3.1(+)-Myogenin and pcDNA3.1(+)-MEF2A, 2 µL of Lipofectamine 2000 using Lipofectamine 2000 (Invitrogen Corporation, Carlsbad, NM, USA) according to the manufacturer’s protocol, and the pcDNA3.1 vectors were cotransfected with pGL3-basic-ucp3-P1, which was the control. After 5 h, the OPTI-MEM medium was replaced by DMEM medium containing 10% FBS. After 24 h transfection, luciferase activity was measured using the same techniques as those described for C2C12 myoblasts cells.

### 4.8. The Data Analysis

Statistical analyses were performed using SPSS17.0. Data (SPSS statistics 17.0, WinWrap Basic, New York, NY, USA, 2008) were presented as the mean ± standard error of the mean. Statistical differences between conditions were assessed using one-way ANOVA, *p* < 0.05 was considered statistically significant.

## 5. Conclusions

The results showed that *UCP3* promoter led to tissue-specific expression in the muscle. The activity of six different lengths of *UCP3* promoter were determined using the dual luciferase reporter gene. The results suggest that there may be positive regulatory elements in the region of the *UCP3* promoter from −433 to −385 bp, and there may be negative regulatory element in the region from −620 to −433 bp. Double luciferase assay and PEGFP-N3 recombinant vector results suggested that the region of *UCP3* promoter from −385~+3 bp may be the core promoter region. We successfully screened out that the *UCP3* promoter region of Guanling cattle have transcription factor binding sites which include *MyoD*, *TFIID*, *Pax3*, *MEF1* and *MEF2* binding sites. Co-transfection experiments of the transcription factors found that *Myf5*, *Myf6* and *MyoD* have a certain positive regulatory role with regard to activity of the *UCP3* promoter. The result indicated that the *UCP3* promoter may play an important role in the muscle tissue.

## Figures and Tables

**Table 1:** Sequence prediction results of uncoupling protein 3 (*UCP3)* promoter.

Start	End	Score	Promoter Sequence (5′ to 3′)
−907	−857	0.95	ATTAAAATGCATGAATCCGCAGTCATAAAAAGGCACGAAGTGCTGACATA
−842	−792	0.87	AACTTGGAAAACACTTTGGTAAGTAAAAGAAGCCAGTCACAAAAGGTCA
−449	−399	0.80	GAATTAGCATCACCATTGTTACTATTAAGAGGAGGCCCAGGGGAACCCTG
−301	−251	0.99	TCATCACCTGCAATGGGGTGTTTTAAAGAAGCCCAAGTCAAGGGAACTGA
−154	−104	1.00	AAGGTTTCAGGTCAGCCAGAGTGTATAAAGACAGGTGCCAAGCCAGAGGC
−52	−2	1.00	CCTGGGATGGAGCCCTGAGGACCCTTAAAGAGGCCCCATGCTTCCGCCAC

**Table 2:** The prediction results of transcription factor binding sites of *UCP3* gene promoter in Guanling cattle by online software TFSEARCH.

Site	Transcription Factor Binding Sites
−1300~−1200 bp	*GATA-1*, *p300*, *MZF1*
−1200~−1100 bp	*GATA-1*, *GATA-2*, *GATA-3*, *GATA-X*, *C/EBPb*, *C/EBP*, *MZF1*
−1100~−1000 bp	*CdxA*, *Nkx-2*, *AML-1a*, *GATA-1*, *GATA-2*, *GATA-3*, *GATA-X*, *Cdx*, *COUP-T*
−1000~−900 bp	*NF-kap*, *Lyf-1*, *CdxA*, *NF-kap*, *Sox-5*, *GATA-1*, *Pbx-1*
−900~−800 bp	*CdxA*, *C/EBP*
−800~−700 bp	*GATA-X*, *GATA-2*, *CdxA*, *SRY*, *Egr-1*, *Egr-2*, *Egr-3*, *NGFI-C*
−700~−600 bp	*MZF1*, *Oct-1*, *GATA-1*, *CdxA*, *AML-1a*, *Nkx-2*
−600~−500 bp	*CdxA*, *C/EBP*, *USF*, *Nkx-2*, *SRY*, *C/EBPb*, *HNF-3b*, *S8*, *HFH-2*
−500~−400 bp	*Sox-5*, *GATA-1*, *S8*, *Oct-1*, *Nkx-2*, *CdxA*
−400~−300 bp	*CdxA*, *GATA-X*, *AML-1a*, *HNF-4*, *GATA-1*, *deltaE*, *C/EBPb*, *USF*, *Lyf-1*
−300~−200 bp	*TATA*, *SRY* , *AML-1a*, *Nkx-2*, *USF*, *Sp1*
−200~−100 bp	*Nkx-2*, *USF*, *deltaE*, *CdxA*, *TATA*, *MyoD*
−100~+1 bp	*Nkx-2*, *USF*, *deltaE*, *CdxA*, *TATA*, *MyoD*
+1~+100 bp	*GATA-1*, *GATA-2*, *CdxA*

**Table 3:** Sequences of primers used for real-time PCR.

Gene Symbol	Forward Primer (5′ to 3′)	Reverse Primer (5′ to 3′)	Length (bp)
*β-actin*	GCAGGTCATCACCATCGG	GCTGTCACCTTCACCGTTC	564 bp
*RPL4*	AGGAGGCTGTGTTGCTTCTG	CGACGGTTTCTCATCTTGC	106 bp
*18SRNA*	GACTCAACACGGGAAACCTC	AGACAAATCGCTCCACCAAC	120 bp
*UCP3*	TCTACGACTCCGTCAAGC	CTGGCGATGGTCCTGT	469 bp

**Table 4:** Sequences of primers of *UCP3* promoter used for PCR.

Gene Symbol	Forward Primer (5′ to 3′)	Reverse Primer (5′ to 3′)	Length (bp)	Tm (°C)
*UCP3*-P1	CTAGCTAGCTACCCTCAGTGCCTATCA	CCGCTCGAGGCCATTTGCTGGTGTCT	1080	56
*UCP3*-P2	CTAGCTAGCGCCTCCAAGTGTCATGT	CCGCTCGAGGCCATTTGCTGGTGTCT	980	60
*UCP3*-P3	CTAGCTAGCTGAATCCGCAGTCATAAA	CCGCTCGAGGCCATTTGCTGGTGTCT	814	60
*UCP3*-P4	CTAGCTAGCTGAAGGGAAGAGGAAACA	CCGCTCGAGGCCATTTGCTGGTGTCT	624	59.4
*UCP3*-P5	CTAGCTAGCCCTACCTCGTAAGAGTTGTG	CCGCTCGAGGCCATTTGCTGGTGTCT	437	60
*UCP3*-P6	CTAGCTAGCCACATAGTAGGCACCAAGA	CCGCTCGAGGCCATTTGCTGGTGTCT	389	59.3

**Table 5:** The system of experimental group and control group.

Reagent	Experimental Group (μL)	Compare Group (μL)
TF Binding buffer mix	15	15
TF Probe mix	3	3
*UCP3* promoter PCR fragment	5	0
Nuclear extract	6	6
ddH_2_O	1	6
Total Volume	30	30

**Table 6:** Sequences of primers of coding sequence of MRFs family and myocyte-specific enhancer factor 2A (*MEF2A*) used for PCR.

Gene Symbol	Forward Primer (5′ to 3′)	Reverse Primer (5′ to 3′)	Length (bp)
*MyoD*	EcoRI: CCGGAATTCATGGAGCTGCTGTCGC	XhoI: CCGCTCGAGTCAGAGCACCTGGTAAAT	975
*Myf5*	EcoRI: CCGGAATTCATGGACATGATGGACGGCTG	XhoI: CCGCTCGAGTCATAGCACATGATAGATG	786
*Myf6*	KpnI: CGGGATCCATGATGATGGACCTTTTTGAAACTGGC	EcoRI: CGGAATTCTTACTTCTCCACCACCTCCTCCACGCAG	745
*MyoG*	BamHI: GGGGTACCATGGAGCTGTATGAGACCTCT	EcoRI: CGGAATTCTCAGTTTGGTATGGTTTCATCTGG	691
*MEF2A*	EcoRI: CCGGAATTCATGGGGCGGAAGAAAATACAA	XhoI: CCGCTCGAGGTTAGGTCACCCACGCATC	1498
